# Alcohol Warnings and Moderate Drinking Patterns among Italian University Students: An Exploratory Study

**DOI:** 10.3390/nu9060628

**Published:** 2017-06-17

**Authors:** Azzurra Annunziata, Riccardo Vecchio, Angela Mariani

**Affiliations:** 1 Department of Economic and Legal Studies, University of Naples Parthenope, 80133 Naples, Italy; angela.mariani@uniparthenope.it; 2 Department of Agricultural Sciences, University of Naples Federico II, 80055 Portici (Na), Italy; riccardo.vecchio@unina.it

**Keywords:** alcohol warning, moderate drinking, young adults, factor analysis, cluster analysis

## Abstract

The introduction of health warnings on labels to correct externalities associated with alcohol consumption is heavily debated and has been explored from different perspectives. The current paper aims to analyse the interest and attitudes of Italian university students regarding health warnings on alcoholic beverages and to verify the existence of segments that differ in terms of attitudes towards such warnings. Our results show that young consumers consider health warnings quite important, although the degree of perceived utility differs in relation to the type of warning. Cluster analysis shows the existence of three groups of young consumers with different degrees of attention and perceived utility of warnings on alcoholic beverages, but also in relation to drinking behaviour and awareness of social and health risks related to alcohol consumption. In brief, Italian young adults with moderate consumption behaviour view label warnings positively, while this attitude is weaker among younger adults and those with riskier consumption behaviours. Our findings, albeit limited and based on stated and not revealed data, support the need for appropriate tools to improve the availability of information among young adults on the risks of excessive alcohol consumption and increased awareness of the importance of moderate drinking.

## 1. Introduction

According to the World Health Organization, alcohol is the third leading risk factor for disease and mortality in Europe, and harmful use of alcohol accounts for nearly 6.5% of all deaths in Europe [1]. Alcohol consumption by young people is cause for increasing concern in European countries due to numerous negative short-term and long-term effects, comprising social, physical, psychological, and neurological consequences reaching into adult life. At the same time, binge drinking, or heavy episodic drinking, is still significantly increasing in some European Union (EU) Member States, while other Member States have witnessed a decrease in such alcohol abuse.

According to the European School Survey Project on Alcohol and Other Drugs (ESPAD), in 2015, more than 35% of young Europeans reported heavy episodic drinking in the past month, while over three-quarters of respondents (78%) reported relatively easy access to alcohol [2]. In Italy, according to the latest statistics published by Italian National Institute of Statistics (ISTAT) (2015) [3] alcohol consumption behaviour that exceeds health recommendations affects more than 15% of the population. The population most at risk are the under 30 s, as 14.5% of young individuals indulge in binge drinking mostly during social events [3].

The recommendations of the WHO Global Strategy to reduce harmful use of alcohol [4] include providing consumer information about, and labelling of, alcoholic beverages to indicate the harm related to alcohol. Nevertheless, while a number of countries have introduced mandatory warning labels on alcoholic beverages, in Europe, a voluntary and unregulated approach still prevails. In this regard, many public health and consumer associations are urging implementation of mandatory health warning labels on alcoholic beverages. At the same time, especially in recent years, alcoholic beverage producers have started to promote several initiatives to voluntarily place health warnings on labels. In this regard, the Beer, Wine and Spirits Producers’ Commitments 2013–2017 include adding to packaging a standard set of easily understood symbols or equivalent words to discourage drinking and driving, consumption by those under age, and consumption by pregnant women [5]. However, the last available report produced in European countries shows that, overall, fewer than one in five alcohol labels (17%) contained a health-related message in addition to the mandatory alcohol content information in each country [6]. Furthermore, several studies show that recognition of alcohol warning labels is limited when warnings are voluntarily placed by the alcohol industry, as they tend to be text-based, indirect, vague, and barely visible [7,8]. Much has been written on the impact of the introduction of alcohol warning labels on consumer attitudes, knowledge, and behaviour, suggesting that alcohol warning labels may improve knowledge and attitudes regarding the harmful consequences of alcohol use among adults [9,10,11]. Other studies have been carried out to explore consumer reactions to alternative formats on warning labels, highlighting the importance of framing and the emotional appeal of a health warning [8,12,13]. However, most of these studies were performed in the United States or in Australia, while the interests and attitudes of European consumers have not yet been fully explored [14,15,16,17,18]. Furthermore, few scholars have analysed the relative influence of alcohol warning statements on youth choices (individuals under 30 years old).

Against this background, the current paper intends to contribute to the existing literature by presenting the results of an empirical study on the interest and attitudes of Italian university students towards health warnings on alcoholic beverages. In addition, the paper aims to verify the existence of segments of young consumers that differ in relation to their attitudes to health warnings and their preference for alternative formats in order to provide useful indications to tailor private and public initiatives. More specifically, the following research questions are investigated: (i) are Italian university students interested in receiving warnings on alcoholic beverage labels? and (ii) are there distinct segments of university students with different attitudes towards warning labels and different preferences? The contribution of the current paper to the existing literature is twofold. First, it sheds some light on young adults living in Europe, and especially in Italy, where the topic has not yet been fully explored [14,15,16]. Secondly, this paper provides market segmentation, which is widely recognized as a core device for tailored public education and communication campaigns.

## 2. Research Background

Health warnings are widely considered a useful way to inform consumers on risks associated with alcohol and can also potentially reduce dangerous drinking behaviour by increasing consumer awareness of the harmful effects of alcohol [11,19]. The premise underlying the introduction of such labels is that these warnings would provide a significant symbolic message about the dangerous quality of the product [20]. As suggested by the literature, we may distinguish two complementary ways in which health warnings on alcohol labels may influence consumption behaviour: by targeting individual drinkers at the point of purchase and consumption to influence short-term behaviour; and in the longer term, by providing messages that alcohol is an exceptional and potentially hazardous commodity [20,21].

As previously noted, there is a large body of literature examining the impact of the introduction of alcohol warning labels. Nevertheless, while there is broad consensus that this type of information may improve knowledge and raise awareness and prompt discussion on the harmful health and social consequences of alcohol abuse [10,20], mixed results have been obtained in numerous studies examining the effectiveness of alcohol warning statements in changing consumer behaviour [22]. In particular, studies performed in the United States have found that alcohol warnings have no effect on beliefs about alcohol or intended alcohol-related behaviour [23], while others have found moderate effects on consumption [24]. According to Al-hamdani (2014) [22], the mixed results obtained in previous studies are mainly connected to the weak content of warnings on alcohol products, their poor visibility, and the lack of pictorial content.

With specific reference to the youth cohort, based on the available literature, it appears that younger consumers support the presence of alcohol warning labels on beverage containers [7,15,16,21,25]. Indeed, Stockwell’s (2006) review [10] concluded that awareness of alcohol labels in the United States is highest among young people and heavy drinkers. However, he also concludes that warning labels had no significant impact on drinking behaviour, though associated with increased discussion of the harmful effects of alcohol consumption. In their review, Scholes-Balog et al. (2012) [11] suggested that adolescents may be a particularly good audience for such warning labels as they appear to be more supportive than adults of the warning messages. Annunziata et al. (2016) [14] reveal that among Italian wine consumers, individuals belonging to the lower age groups (18–35) seem more interested in health warnings on the label than older respondents. Coomber and colleagues (2015) [7], in their study on Australian consumers, found that younger adults were also more likely to be aware of the warning labels mainly due to a higher exposure to such messages. Also, MacKinnon et al. (2000) [23] indicated that three to four years after introduction of warning labels in the US, adolescents had increased awareness of labelling laws, exposure to labels and recognition memory of alcohol warning labels. Glock and Krolak-Schwerdt (2013) [24] found that health warnings on alcoholic beverages may specifically influence college-aged people.

It has been widely shown that individual characteristics may affect the impact of alcohol warning labels on drinking behaviour. In this regard, Nohre and colleagues (1999) [25] reported that gender, ethnicity, familiarity with alcohol, socioeconomic status and individual school grades all influenced the impact of alcohol warning labels. The impact is also affected by drinking frequencies and attitudes and awareness about alcohol-related risks. Indeed, Cryer et al. (2002) [26] reported that for both US and Australian students drinking frequencies, i.e., binge or non-binge drinking, strongly affect the perception of different warnings on alcoholic beverages. While Weiss (1997) [27] found that students with extensive knowledge of alcohol-related risks associated with pregnancy and drink-driving, as well as alcohol-related health problems, strongly support the use of alcohol warning labels. Similarly, Annunziata et al. (2016) [14] revealed that consumers with greater concern for drinking issues in modern society were highly interested in alcohol warning labels.

With reference to consumer reactions to alternative formats on warning labels, there is a certain consensus that the use of generic messages is not very effective. By contrast, customised messages seem to be more effective [28]. In addition, increasing the visual salience by using graphic warnings as well as front-of-pack labelling might be more effective in attracting and maintaining attention [29]. In this regard, Jarvis and Pettigrew (2013) [30] revealed that negatively framed health statements had greater impact, especially on groups at risk. Krischler and Glock (2015) [31] demonstrated that warning labels presented as statements had no influence on individual and general outcome expectancies and drinking intentions. Hassan and Shiu (2017) [13] showed that students view pictorial health warnings which encourage low-risk consumption of alcohol as both credible and personally relevant. Wigg and Stafford (2016) [12] found that graphic alcohol warning labels increase arousal, fear, and intention to abstain from alcohol use among university students. Finally, Al-hamdani and Smith (2017) [8] found that graphic warnings enhance warning recognition.

## 3. Material and Methods

### 3.1. Sample Characteristics and Survey Design

**Figure 1:**
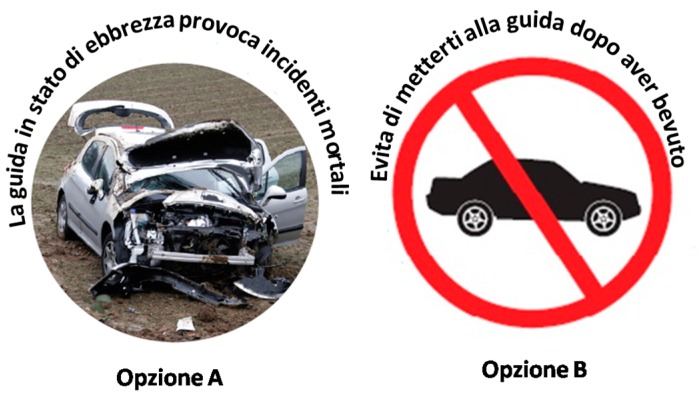
Alternative formats proposed to interviewees. Option (**A**): Drinking while drunk causes fatal accidents; Option (**B**): Do not drink and drive.

A quantitative survey was conducted on a sample of university students, aged between 18 and 30 years old, through a questionnaire administered on line. Student recruitment took place between March and May 2016 at the University of Naples “Parthenope” (Southern Italy), by sending an email inviting individuals to participate in the survey. Over 600 e-mails were sent to students enrolled on undergraduate and graduate courses. A total of 385 completed interviews were collected. The protocol used for data collection complied with national ethical requirements. All data were collected anonymously and recorded according to the Italian Personal Data Protection Code—Law Decree no. 196 of 30 June 2003. All subjects gave their informed consent to participate in the study. Participation was voluntary, without any reimbursement or incentive. The original survey was in Italian. The questionnaire was pilot-tested with a sample of 20 students in order to verify the comprehensibility of questions and the suitability of the language for the target in question. The final questionnaire comprised 30 questions, sub-divided into four sections:

(i) The first section analysed general habits and motivation of alcohol consumption, asking questions previously used in national and European surveys on alcohol consumption behaviour [3,18];
(ii) The second section analysed awareness of health and social risks related to alcohol consumption by using seven statements from previous literature [14,15,16,32], asking consumers to express their agreement on a five-point scale (ranging from 1 = strongly disagree, to 5 = strongly agree). The internal reliability of these items was assessed through Cronbach’s alpha (α = 0.82). Furthermore, knowledge of alcohol content of different beverages and legal limits to drink and drive was tested by using two additional multiple-choice questions;
(iii) The third section aimed to analyse attitudes towards information and health warnings on alcoholic beverages, using questions and statements from previous research (e.g., [30,32]). General attention and visibility of information on labels were tested with reference to specific information that may be currently (voluntarily) reported on alcoholic beverage labels: number of drinks not to exceed; drinking responsibly; and a warning related to drinking and driving (Table 1). However, it should be pointed out that the voluntary use of warnings and information on moderate consumption in Italy is quite limited, as no common or coordinated action between private and public entities has been developed (unlike other countries such as Australia—e.g., the Drinkwise programme—and the United Kingdom—e.g., the drinkaware programme). The degree of perceived utility was assessed by selecting five warnings from the European Alcohol Policy Alliance (Eurocare) library of health warning labels (2012) [33], asking respondents to express their degree of perceived utility of these labels in changing drinking behaviour (ranging from 1 = strongly useless, to 5 = strongly useful). The Eurocare library proposes ten warning statements, of which we selected the five considered to have most relevance to young people in line with the existing literature [30]. Finally, preferences for different warning formats were tested, showing participants two different warning labels related to drinking and driving (Figure 1), differentiated by positive and negative framing, to investigate the stated effect.
(iv) The fourth section collected the socio-demographic characteristics of participants.

### 3.2. Data Analysis

Descriptive statistics were used to report percentages, means, and standard deviations of the main variables. Subsequently, exploratory factor analysis, with the Varimax rotation method, was applied to group different variables that affect consumers’ attention and attitudes regarding health warnings on alcoholic beverages. Based on factor analysis results, the sample was segmented with k-means cluster analysis, classifying the statistical units identified into a set of ‘exclusive and exhaustive’ clusters so as to maximise their internally homogeneous nature and externally heterogeneous nature and identify different consumer profiles. This method was chosen as it is widely used in similar studies that explore alcohol-related attitudes and behaviours (e.g., [34,35,36]). Data were analysed using SPSS version 22.

## 4. Results

### 4.1. Descriptive Analysis

The survey participants were predominantly male (54%), with a mean age of 22.4 years. Of the students surveyed, 58% were attending the first three years of courses, 36% had enrolled in the final two years, while the remaining 6% were graduate students attending a PhD or a Masters course.

#### 4.1.1. General Habits and Motivation of Alcohol Consumption

General alcohol consumption habits are reported in Table 5. It is worth noting that almost half the sample stated that they drank alcohol only on special occasions and drank only one beverage per occasion, while 5% consumed alcohol on a daily basis. Alcoholic beverages were mostly consumed outside the home and between meals (36%). One-third of the sample stated they had drunk excessively to the point of being drunk at least once in the previous month, while almost half never did so. These data are similar to those reported by ISTAT data, in which 60% of the Italian young adult population (between 18–24) drink alcoholic beverages occasionally and 8% daily [3]. National statistics also reveal that the places where young adults tend to consume alcoholic drinks are friends’ houses (41.8%), bars or pubs (27.4%), restaurants and pizzeria (24.4%), and discotheques or night clubs (13.3%), and it also reveals that, especially among young men, the incidence of those consuming alcohol between meals has increased in recent years, reaching 43.2% among 18–34 years old [3].

As for the reasons that lead young adults to consume alcoholic beverages (as shown in Table 4) the hedonistic motivation linked to the taste of the drink was considered the most important (M 3.28), followed by the motivation “it is fun” (M 2.88) or “to relax” (M 2.34).

#### 4.1.2. Awareness of Health and Social Risks Related to Alcohol Consumption

The spread of alcohol consumption among young individuals is considered a fairly significant problem by 60% of the sample. As reported in Table 4, respondents revealed great awareness of the risks of developing an addiction, as well as the risks of altering driving ability and the impact of alcohol on mood disturbance and on concentration ability. Less awareness is shown vis-à-vis the contribution of alcohol to being overweight and obese, the risks of drinking while taking medicine, and drinking during pregnancy. Three-quarters of respondents considered it necessary to receive further information on the risks related to excessive alcohol consumption mainly from public campaigns on TV (21%) or the social media (17%). Labelling is indicated as an ideal tool to obtain more information in only 13% of cases.

#### 4.1.3. Attitudes to Information and Health Warning on Alcoholic Beverage Labels

Almost half the interviewees (48%) paid sporadic attention to the information on the labels of alcoholic drinks and 28% never read the labels on alcoholic beverages. Respondents considered that there was too much information and too many warnings on labels and, therefore, they tended to ignore them (M 3.7, SD 0.92) and rated them as unimportant (M 3.4, SD 1.04).

Table 1 shows the results of attention and exposure effect of some voluntary information placed by companies on alcoholic beverage labels. The number of glasses not to exceed represents the least visible information, since only 7% of respondents claimed to have noticed it at least once, and most of those who noticed the labelling stated they had not changed their behaviour in any way.

The statement “drink responsibly” has more visibility (32% stated they had noticed it at least once), but also in this case for most respondents this information did not affect behaviour. The warning logo “do not drive after drinking” has the highest visibility (27% of respondents claimed they had noticed it often). In terms of the exposure effect, half the sample stated that the information did not have any influence on their behaviour; while 19% revealed they avoided driving after drinking and 16% said they had reflected on the potential risks of drinking and driving.

Considering the five specific warnings selected from the Eurocare library (Table 2), on average, respondents found the following recommendations more useful: do not drink while taking medicine and do not drink and drive. Lower utility is assigned to the more generic recommendation don’t serve alcohol to underage individuals as well as the indications of long-term effects related to potential harm during pregnancy and potential damage to brain functions.

As regards the respondents’ reactions to alternative formats on pictorial warning labels (Figure 1), our results show that 82% of the sample attach more emotional impact to the negative framed warning (logo with the wrecked car) rather than the generic non-emotive symbol. It is also important to highlight, however, that the text associated with the negative framed logo can be considered more direct and specific than that associated with the alternative option.

### 4.2. Segmentation Analysis

The sample was segmented to ascertain the existence of homogeneous groups of consumers with different propensities towards health warnings on alcoholic beverages. Thus, a factor analysis, using the principal component method, was first performed to reduce the variables related to attention to, and perceived utility of, information on labels to two factors. The maximum likelihood method with varimax rotation was applied to extract the theoretical factors. Varimax rotation with Kaiser normalization is the method employed almost exclusively when performing orthogonal rotation (Kaiser, 1958) [37]. Both the scree plot and initial eigenvalue test were used. The factor structure and factors’ internal reliability are summarised in Table 3. These two factors together explain 62% of the original variance. Both attention (α = 0.67) and perceived utility (α = 0.77) had sufficient internal reliability consistency, as confirmed by Cronbach’s alpha.

**Figure 2:**
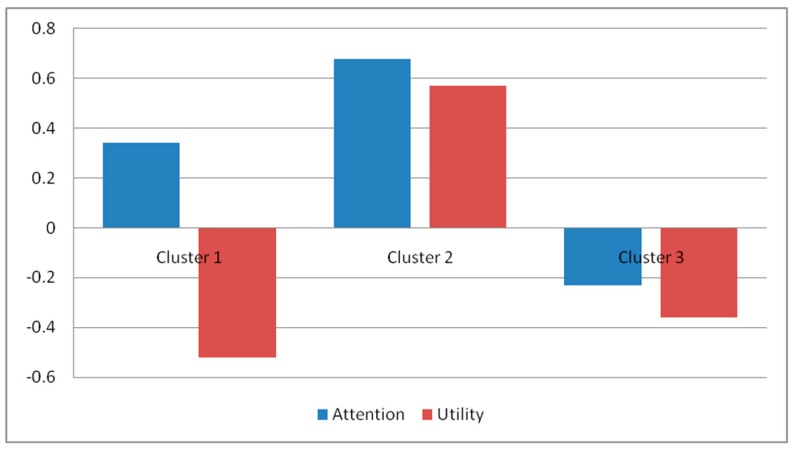
Final cluster centre.

Based on the factors identified, non-hierarchical clustering (with the K-means method) was performed to obtain segments. Bivariate analyses including cross-tabulation with χ^2^-statistics, independent samples *t*-test and one-way ANOVA comparison of means were then used to profile each cluster. From the application of this method, it was found that division into three groups was the ideal solution. As shown in Figure 2, the identified clusters differ in relation to the degrees of attention and perceived utility of warnings on alcoholic beverages. One-way ANOVA also reveals the existence of significant differences between the groups in relation to variables linked to drinking motivation, information, and awareness of the social and health risks associated to alcohol consumption and general attitudes towards warnings (Table 4). Finally, differences related to general habits of alcohol consumption and socio-demographic variables between clusters were investigated using cross-tabulation and χ^2^ association tests (Table 5).

**Table 4:** Cluster characterisation (mean scores).

	Cluster 1 *Doubting* (32%)	Cluster 2 *Responsible* (40%)	Cluster 3 *Uninformed* (28%)	Total Sample
*Awareness of alcohol-related risk*				
How well-informed do you consider yourself on the risks related to excessive alcohol consumption?	3.5 ^a^	3.7 ^a^	2.8 ^b^	3.3
Alcohol can cause addiction	4.1	4.4	4.1	4.2
Alcohol consumption contributes to being overweight and obese	3.2 ^a^	4.1 ^b^	3.0 ^a^	3.4
Alcohol consumption alters one’s driving ability	4.1 ^a^	4.4 ^b^	4.0 ^a^	4.1
Alcohol consumption alters one’s ability to concentrate	4.0 ^a^	4.3 ^a^	3.8 ^b^	4.0
Alcohol consumption alters one’s mood	4.0 ^a^	4.4 ^b^	3.8 ^a^	4.0
Alcohol must be avoided when taking medicine	3.9 ^a^	4.1 ^a^	3.5 ^b^	3.8
Alcohol must be avoided during pregnancy	3.7 ^a^	4.2 ^b^	3.5 ^a^	3.8
*Drinking motivation*				
It is fun	2.8 ^a^	2.6 ^a^	3.3 ^b^	2.9
I like the taste of alcoholic beverages	3.2 ^a^	3.8 ^b^	3.0 ^a^	3.3
I drink as all my friends do	1.7 ^a^	1.9 ^a^	2.8 ^b^	2.1
I drink to relax	2.4	2.3	2.5	2.3
*General attitudes towards warnings*				
Warnings are not relevant to me	3.7 ^a^	2.8 ^b^	3.9 ^a^	3.4
There are too many warnings on labels	3.9 ^a^	3.4 ^b^	3.8 ^a^	3.7
*Perceived utility*				
Utility assigned to maximum number of glasses not to exceed	3.6 ^a^	4.2 ^b^	3.2 ^c^	3.6
Utility assigned to drinking responsibly	3.7 ^a^	3.9 ^a^	2.8 ^b^	3.4
Utility assigned to not drink and drive	3.9 ^a^	4.3 ^b^	3.0 ^c^	3.7

Different superscripts indicating group means differ at least at the 10% significance level using the Bonferroni test.

**Table 5:** Cluster profiles.

		*Doubting*	*Responsible*	*Uninformed*	*Total*	*Sig.*
Age group	18–21	30	25	36	*38*	*0.006*
	22–25	36	21	30	*32*	
	26–30	34	54	34	*30*	
Education Level	Attending first three years	55	46	61	*58*	*0.053*
	Attending final two years	21	29	20	*36*	
	Attending Masters/PhD	24	25	19	*6*	
Gender	Female	40	54	31	*44*	*0.000*
	Male	60	46	69	*54*	
Frequency of alcohol consumption	Only on special occasions	16	44	39	*47*	*0.001*
	Once a week	39	34	22	*30*	
	Two/three times a week	36	19	29	*18*	
	Every day	9	3	10	*5*	
Number of drinks per occasion	One	50	56	42	*50*	0.010
	Two	32	30	24	*31*	
	Three	11	13	23	*13*	
	>Three	7	1	11	*6*	
Place of drinking consumption	Pub or pizzeria	39	42	38	*39*	*0.622*
	Discotheque	26	25	27	*24*	
	Bar	18	21	18	*22*	
	At home	17	12	17	*15*	
Number of times that you have felt drunk in the past year	Never	58	69	49	*53*	*0.020*
	Less than once a month	31	28	30	*33*	
	More than once a month	11	3	21	*14*	
Knowledge of legal limits to drinking and driving	Correct answer	61	64	58	*59*	0.013
	Incorrect answer	11	12	11	*10*	
	Don’t know	28	24	31	*31*	
Knowledge of alcohol content of beverages	Correct answer	48	63	41	*48*	0.036
	Incorrect answer	36	26	39	*36*	
	Don’t know	16	14	20	*16*	
Smoker	Yes	31	27	45	*48*	*0.000*
	No	69	73	55	*52*	

The first cluster groups 32% of respondents who stated that they occasionally paid attention to warnings on labels and at the same time did not consider such warnings to be particularly useful to steer their consumer choices. As regards alcohol consumption habits, this cluster is dominated by young adults who drink once a week (39%) or 2–3 times (36%), mainly consuming one drink per occasion. Just under one-third (31%) of this cluster drink excessively less than once a month, while over 10% do so more than once a month. These individuals drink alcohol mainly because they like the taste or to have fun. Respondents in this cluster also stated they were averagely informed of the risks linked to alcohol consumption, even though compared to some specific aspects such as the relationship between alcohol and obesity and the risks of use during pregnancy their awareness was below the average of the total sample. With regard to the degree of knowledge of the alcohol content of different beverages, most of the individuals in this group were unable to indicate the correct answer (36% were wrong and 16% did not know). Summarising the characteristics described above, this group may be described as the doubting cluster. With respect to socio-demographic variables, 36% were students between 22–25 years and were mostly attending the first three years of university, although about 24% were studying for a Masters course or a PhD.

The second cluster is the largest and accounts for 40% of respondents who show greater attention towards the warnings on the label and a higher perceived utility. These individuals attribute greater importance to the warning on the label, even if they believe that it contains too much information. This segment differs from the others in its level of awareness of the risks linked to alcohol consumption—on average it is higher than that of the other two clusters, especially vis-à-vis the link between alcohol consumption and obesity and the risks of alcohol use during pregnancy. Awareness of the legal limits to drinking and driving found in this group are also higher than that of the total sample average: over 64% of the individuals in this group provided the correct answer, as they did for their knowledge of the alcohol content of drinks (over 60% responded correctly). With regard to the drinking habits of interviewees, individuals in this cluster drink alcohol less often, only on special occasions (44%) or once a week (34%), mainly consuming one drink per occasion. Almost 70% of individuals in this cluster never felt drunk during the last year. Given the characteristics described above, this group may be termed the responsible cluster. Focusing on the socio-demographic variables of this cluster, most were between 26 and 30 years old (54%), with 29% enrolled in the final years of university or doing a Masters course or PhD (25%).

Finally, the third cluster, which includes 28% of the sample, is characterised by a lack of attention to warnings on labels, but also by a low level of perceived utility of warnings in changing consumer behaviour. Compared to the other two clusters, this cluster comprises less well-informed individuals with lower awareness of the social and health risks associated with alcohol consumption. Young adults in this cluster drink alcohol more frequently than those in the other two clusters (2/3 times a week in 29% of cases or every day in 10% of cases). They also stated they consumed more drinks per occasion (11% over three drinks) and reported feeling drunk with greater frequency (over 20% more than once a month). Furthermore, in this cluster, there is a higher concentration of respondents who said they drank to imitate their friends and to have fun. Respondents in this cluster show limited knowledge of the alcohol content of different alcoholic beverages and of the legal limits to driving after drinking (more than 31% were unaware of this information). For these reasons, this segment can be called the uninformed cluster. As regards the socio-demographic profile of this cluster, it includes younger individuals (36% aged between 18 and 20), mostly men, who are attending the first three years of university (61%). Cluster 3 also contains a higher proportion of smokers (45%).

## 5. Discussion

The current study explored the interest and attitudes of Italian university students (*N* = 385) concerning health warnings on alcoholic beverages and verified the existence of consumer segments with different attitudes and preference to health warnings. Our findings show that young adults are reasonably aware of the risks linked to excessive alcohol consumption and consider the spread of alcohol consumption among their age cohort a serious matter. In addition, respondents would like to have further information, preferring public campaigns on TV or on social media and warning posters in places licensed to sell alcoholic beverages. Information on bottle labels represents the least preferred source of information: in most cases such labels are considered excessive by respondents and are read only occasionally. These findings are in line with results from a survey conducted in the context of the European Union’s Joint Action on Reducing Alcohol Related Harm (RARHA) among individuals under 30 across 21 European countries [38]. Moreover, the scant attention devoted to the information on alcoholic drink labels is consistent with the fact that most of the respondents consume alcoholic beverages outside the home, in places where such products are served directly in glasses, as pointed out by other studies [39,40]. However, other studies carried out in Anglo-Saxon countries (e.g., Australia) revealed that most young individuals consumed alcohol directly from a can or bottle, and that this consumption mode has a significant positive association with support for warning labels [7].

On the other hand, our results highlight the fact that respondents are confused on the concept of moderate alcohol consumption and have scant knowledge of the thresholds imposed by the law to limit driving after drinking. Furthermore, there emerges a problematic evaluation of alcohol content per drink. Also in this case, our results are in line with the RARHA report [38] which points to confusion and misunderstanding regarding the concept of alcohol content per drink, standard drinking, and low-risk drinking, especially among the younger segment.

Findings also show that young individuals consider health warnings quite important, although the degree of perceived utility differs in relation to the specific type of warning. Higher utility is attached to the warning avoid drinking if you are taking medicine and do not drink and drive while less utility is attached to warnings concerning the potential long-term effects of alcohol, such as brain damage or maternal and foetal risk during pregnancy. Similar results were found by previous research [39,40,41] concluding that Australian university students may consider warning labels ineffective because they do not perceive themselves to be personally vulnerable to the long-term consequences of alcohol use, or do not perceive such consequences to be relevant to them at this point in their lives, attaching more weight to the short-term than long-term consequences of their decisions. In addition, we reveal that young individuals attach more emotional impact to the negative framed warning (logo with the wrecked car) rather than the generic ones, confirming results from previous research [8,22].

Nevertheless, our findings also reveal that the level of visibility of warnings currently voluntarily present on the bottles is very low, as is their effectiveness in changing consumption behaviour. This is in line with results from Coomber et al. (2015) [7], who show that for Australians, the rate of recall of both the ‘Get the facts’ logo and warning labels voluntarily introduced is very low, even if their results suggest that younger respondents were also more likely to be aware of the warning labels than older individuals. Similarly, Kersbergen and Field (2017) [29], using eye-tracking, showed that students from the University of Liverpool allocate minimal attention to warning labels voluntarily placed on alcohol packaging and that even when participants had attended to them, their drinking intentions were not affected.

However, it is important to underline that the difference in the degree of support for warning labels could be linked to the differences in the extent of drinking behaviour existing among countries and also in differences in precisely where and how alcohol is consumed. Several studies have demonstrated that drinking patterns of young people in Mediterranean and Anglo-Saxon countries vary greatly and are influenced also by cultural differences [42]. In particular, as reported by Italian National Statistics data [3], in Italy, the behaviour of drinking before going out is not widespread. Such behaviour seems to be more common in other countries [43]. Furthermore, our results confirm that even when young adults claim to have seen the warning on the label, the presence of the warning does not affect their drinking behaviour, but in some way encourages thought or debate.

Cluster analysis proved the existence of three groups of young individuals which differed in their degrees of attention and perceived utility of warnings in alcoholic beverages, but also in relation to drinking behaviour and awareness of social and health risks of alcohol consumption. University students with a responsible approach towards alcohol (e.g., cluster 2), who drink less often (mainly on special occasions) and are more aware of the social and health risks, reveal a positive attitude towards warnings and a greater level of attention towards currently available, voluntary warnings. The group of young adults with a moderate consumption pattern, who drink for pleasure and fun and are not fully informed on risks (e.g., cluster 1), only occasionally pay attention to warnings on labels and are doubtful about their utility in influencing their choices. Lastly, the youngsters with both higher drinking frequency and heavy drinking behaviour, mainly motivated by imitating friends and are uninformed about social and health risks associated with alcohol consumption (e.g., cluster 3), do not pay attention to warning labels and perceive them as being of little use or relevance. Overall, in line with Scholes-Balog et al. (2012) [11], it may be stated that individual characteristics of young adults may moderate the impact of alcohol warning labels as well as the differences in their alcohol consumption behaviour.

In brief, our results extend previous findings, highlighting that young adults with a moderate consumption behaviour possess a positive attitude towards label warnings, while this attitude and support is weaker among younger adults and those with riskier consumption behaviours. Indeed, our results also confirm the evidence provided by previous research that individuals with a higher alcohol consumption frequency are least interested in health warnings on labels [14,15,16,26,27,28,29]. Considering the third cluster, our results also substantiate findings reported in other research [44,45] that peer pressure plays an important role in influencing alcohol use among youth, as confirmed by the high incidence of respondents who stated they drink to imitate their friends.

## 6. Limitations and Future Directions

Finally, we should underline the possible major limitations in generalising the current results. First of all, the relatively small sample size and geographical coverage (respondents from only one university) seriously limit the representativeness of our participants. Therefore, to enhance the validity of our results, similar studies should be undertaken in other Italian universities with larger (representative) samples. Furthermore, specific shortcomings are intrinsically related to the type of questionnaire format (self-reported) which, albeit anonymous, is prone to social-desirability bias. Additional studies could avoid these limitations by including specific precautions, such as using indirect questioning or applying forced-choice items and possibly using incentive-compatible valuation mechanisms (e.g., non-hypothetical experimental auctions) in which respondents make consequential bids with real products and real money.

In addition, with reference to the alternative formats of pictorial warnings proposed to interviewees (showed in Figure 1), such warnings were proposed on the back label of alcohol bottles. However, this may generate an issue of realism: when such warnings are voluntarily present on the bottle, they are generally located on the bottle’s neck (especially for beer). Based on these limitations, future research should, therefore, focus on identifying the most effective designs to attract people’s attention on warning labels, using for instance eye-tracking methods or developing more realistic labels. Further advances in the present research should also investigate young individuals’ reactions to alternative formats and content of warning labels with a similar experimental design to ensure that the type of warning is not impacting the results. In addition, given that cultural differences affect alcohol-related behaviours and that alcohol warning labels could have differential effects in diverse cultures, future research should be performed on a cross-country sample.

## 7. Conclusions

Given the health and social consequences of alcohol consumption among young adults, interventions designed to promote moderate drinking might be particularly important for improving collective well-being [9]. Alcohol warning labels are intended to increase knowledge and attitudes regarding the harmful consequences of alcohol use and promote changes in consumption behaviour [20]. Although there is no substantial evidence in the literature on the effectiveness of alcohol warning labels in changing behaviour, especially among groups at risk [22,23,24,46], previous research revealed that this could be due to narrow implementation, to the generic nature of the messages used, and to their poor visibility [21,22,28,46]. In this regard, in order to increase the likelihood of alcohol warning messages being perceived as relevant, they need to be congruent with the beliefs of the target audience [9,28]. As suggested by Al-hamdani [22], with a view to enhancing the effectiveness of the warnings, policy makers and private companies should develop more direct health warnings, increase the visibility of the warnings, incorporate pictorial health warnings, and take the entire packaging of alcohol products into consideration.

In conclusion, our findings, albeit limited and based on stated and not revealed data, support the need for appropriate tools to improve the availability of information among young adults about the risks of excessive alcohol consumption and increase awareness of the importance of moderate consumption. At the same time, however, our results show that young individuals who are more attentive to warnings and show greater interest in receiving additional information, are those that are already responsible consumers. These results confirm that warning labelling appears least likely to influence behaviour amongst those that would benefit most from a reduction in drinking. Thus, in accordance with previous research, to improve the overall impact of warning labelling, salient messages with greater force for young adults should be included, such as pictorial warnings tailored to the characteristics and age of this consumer cohort [9,10,11,12,22,33,34,35]. Moreover, in accordance with Argo and Main [47], given that in most cases young drinkers do not see the label on the container, as alcohol is generally consumed in a glass, warning posters in places licensed to sell alcoholic beverages could be more effective.

## Figures and Tables

**Table 1:** Degree of visibility and exposure effect of voluntary information reported on labels.

Voluntary Information on Labels	Attention (% of Total Sample)		Exposure Effect (% of Individuals Who Reported Noticing the Information on the Label)	
Number of glasses not to exceed	Never	91	I have reduced consumption	-
	At least once	7	I have reflected about the effects of alcohol on health	1
	Rarely	2	I have discussed it with friends	2
	Often	-	It does not affect my drinking habits in any way	97
“Drink responsibly” statement	Never	54	I have reduced consumption	4
	At least once	32	I have reflected about the effects of alcohol on health	9
	Rarely	14	I have discussed it with friends	7
	Often	-	It does not affect my drinking habits in any way	80
Warning logo “do not drink and drive”	Never	24	I avoid driving after drinking	19
	At least once	14	I have reflected on the potential risks of drinking and driving	16
	Rarely	35	I have discussed it with friends	15
	Often	27	It does not affect my drinking habits in any way	50

**Table 2:** Degree of utility assigned to different health warnings.

	Mean	Standard Deviation
Do not drink and drive	3.7 ^a^	1.41
Do not drink while taking medicine	3.8 ^a^	1.54
Don’t serve alcohol to underage individuals	2.8 ^b^	1.50
Alcohol can cause harm during pregnancy	2.9 ^b^	1.29
Alcohol can damage brain functions	2.8 ^b^	1.30

Means with different subscripts differ statistically (*p* < 0.05) based on the pairwise comparison *t*-test.

**Table 3:** Matrix of rotated components.

	Attention	Perceived Utility	Communality
Have you ever noticed the following symbols?	0.531	−0.191	0.741
Have you ever noticed the recommendation “*Drink responsibly*”?	0.604	−0.116	0.802
Generally, do you pay attention to the information on labels?	0.339	0.025	0.609
Have you ever noticed the indication of maximum number of glasses not to exceed on the label?	0.462	0.013	0.592
How useful do you consider the warning “*alcohol can cause harm during pregnancy*” on the label?	0.319	0.725	0.713
How useful do you consider the warning “*Don’t serve alcohol to underage individuals*” on the label?	0.219	0.842	0.564
How useful do you consider it to report “*Do not drink while taking medicine*” on the label?	0.250	0.846	0.698
How useful do you consider it to report “*Do not drink and drive*”on the label?	0.298	0.809	0.719
How useful do you consider it to report “*Drink responsibly*” on the label?	0.265	0.774	0.801
How useful do you consider it to report the maximum amount of glasses not to exceed on the label?	0.301	0.672	0.742
There are too many warnings on labels, so I tend to ignore them	0.121	0.763	0.668
Warnings are useful	0.212	0.673	0.725
*% Variance*	*25.21*	*36.97*	
*Cronbach’s alpha*	*0.67*	*0.77*	

Extraction method: Principal Component Analysis. Rotation method: Varimax with Kaiser normalization.

## Abbreviations

The following abbreviations are used in this manuscript:

WHO	World Health Organization
EUROCARE	European Alcohol Policy Alliance
ESPAD	European School Survey Project on Alcohol and Other Drugs
ISTAT	Italian National Institute of Statistics
